# Effects of Acidulants on the Rheological Properties of Gelatin Extracted from the Skin of Tilapia (*Oreochromis mossambicus*)

**DOI:** 10.3390/foods11182812

**Published:** 2022-09-12

**Authors:** Qijia Zhou, Zhiping Zhang, Yiqun Huang, Lihong Niu, Junjian Miao, Keqiang Lai

**Affiliations:** 1 College of Food Science and Technology, Shanghai Ocean University, No. 999 Hucheng Huan Road, Lin Gang New City, Shanghai 201306, China; 2 School of Food Science and Biological Engineering, Changsha University of Science & Technology, Changsha 410004, China; 3 School of Food Engineering, Ludong University, No. 186 Middle Hongqi Road, Yantai 264025, China; 4 Engineering Research Center of Food Thermal Processing Technology, Shanghai Ocean University, Shanghai 201306, China

**Keywords:** fish gelatin, lactic acid, citric acid, malic acid, rheology property

## Abstract

This study aimed to evaluate the effects of lactic acid (LA), citric acid (CA), and malic acid (MA) varying in concentration (0.5–2.0% *w*/*w*) on the rheological properties of fish gelatin (1.5–6.67% *w*/*w*) obtained from the skin of tilapia (*Oreochromis mossambicus*). The addition of LA, CA, or MA in gelatin dispersions significantly (*p* < 0.05) weakened their gel strengths, leading to a 14.3–62.2 reduction in gel strength. The gel strength, elastic (G′), and viscous (G″) moduli, as well as the gelling (T_G_) and melting (T_M_) temperatures of gelatin dispersions decreased with an increased level of acid added, implying the weakening effects of these acids on junction zones of the gelatin network in aqueous media. The addition of LA had less effect on these rheological properties of gelatin dispersions as compared to that of MA and CA, which were consistent with their effects on the pH of gelatin dispersions. Moreover, the reductions of T_G_ and T_M_ for gelatin dispersions with a higher gelatin concentration (e.g., 6.67% gelatin with 0.5% LA, T_G_ dropped 0.4 °C) due to the addition of LA, CA, or MA were less pronounced compared to those with a lower gelatin content (e.g., 2% gelatin with 0.5% LA, T_G_ dropped 7.1 °C), likely attributing to the stronger buffering effect of the high gelatin dispersion and less percentage reduction in the junction zones in the dispersion due to the addition of an acid. Incorporation of the effects of acids on the linear relationships (*R^2^* = 0.9959–0.9999) between the square of gelatin concentrations and G′ or G″ could make it possible to develop a model to predict G′, G″, phase transition temperatures of gelatin dispersions containing different amounts of gelatin and acid (within the tested range) in the future.

## 1. Introduction

Gelatin is a type of high molecular weight, fat-free, and cholesterol-free natural peptide polymer obtained through moderate hydrolysis of collagen [1]. Due to its excellent physical and chemical properties, gelatin is among the most widely applied food gums to provide a viscous and silky mouthfeel and act as a stabilizer in liquid or semi-liquid foods, to solidify some liquid-base foods (such as set-yogurt), to provide chewy yet melted-in-mouth sensory properties in solid foods (such as gums), to be used as key ingredient for making edible-films or coating materials, and so on [2,3,4,5]. Due to some religious restrictions and health concerns on the commonly used porcine- and bovine-based gelatin, fish-based gelatin has become a suitable option, and its application has been steadily increasing in recent years [2,6,7].

The physical, chemical, and physicochemical properties of gelatin dispersions, including sol and gel states and phase transitions (sol-gel and gel-sol transitions), have been widely studied [8,9,10]. Some studies indicate that the physical and physicochemical properties of gelatin dispersions are greatly affected by the chemical properties of gelatin molecules (mainly including amino acid composition, molecular weight distribution, and structure of the gelatin peptides), the concentration of gelatin, and the presence of other compounds (such as sugars, acids, metal nanoparticles, carbonaceous materials, and minerals) in the gelatin dispersion systems [11,12,13,14,15,16]. Noticeably, the amino acid composition, molecular weight distribution, and structure of the gelatin peptides were mainly dependent on the extraction method and the source of the gelatin [15,17]. For example, fish-based gelatin generally has lower gel strength and results in lower viscosity in aqueous media compared to mammal-based gelatin with the same concentration [5,14]. Fish-based gelatin has been extracted from the skins and bones of a wide range of fish species using various methods, and therefore the physical and chemical properties of fish-based gelatin reported from different studies could be quite different. Since the concept of fish-based gelatin is relatively new compared to the mammal-based gelatin, there is a general lack of study regarding the properties of fish-based gelatin, especially considering the variety of the sources and conditions of raw materials for gelatin extraction (such as fish species, growth stages, farming/harvesting and processing methods, storage conditions, etc.). 

Acidulants, such as lactic acid (LA), citric acid (CA), and malic acid (MA), are widely employed in various types of food mainly to improve sensory properties and extend shelf lives of the products [6,18,19,20]. The effects of acids added or changes of pH on the physical and chemical properties of gelatin dispersions have also been noticed [21,22]. Typically, Choi and Regenstein employed NaOH and HCl to adjust the pH of fish gelatin gels (6.67% *w*/*w*), and found that the gel strength was continuously decreased with a decrease in pH from 8 to 2. However, there is still a lack of systematic study regarding the dynamic rheological properties of fish-based gelatin dispersions as affected by the addition of acidulants. 

Therefore, the objective of this research was to investigate the dynamic rheological properties of tilapia (*Oreochromis mossambicus*) skin gelatin dispersions as affected by three different acidulants, including LA, CA, and MA. Tilapia is among the most harvested freshwater fish throughout the world, providing a relatively large quantity and continuous supply of tilapia skins for extraction of high-quality fish gelatin [2,6]. A better understanding of the effects of various types of acidulants with various concentrations on the physicochemical properties of tilapia skin gelatin would be beneficial for properly applying this protein-based food hydrocolloid in various food applications. 

## 2. Materials and Methods

### 2.1. Extraction of Tilapia Skin Gelatin

The skins of tilapia (*Oreochromis mossambicus*), provided by Xiangtai Aquatic Products Co., Ltd. (Hainan, China), were stored at −80 °C before use. The extraction of gelatin was conducted as described by Niu [2] with minor modification. In short, thawed fish skins (4 °C, 24 h) without scales were cut into 1 × 1 cm^2^ pieces, washed with ice water, and pretreated with NaOH (0.3 M,1:6, *w*/*v*) and acetic acid (0.15 M, 1:8, *w*/*v*) at 4 °C for 1 h. Afterward, the gelatin was extracted in deionized water (1:4, *w*/*v*, 50 °C, 3 h), filtered with a vacuum pump filter, and dehydrated at 50 °C in a drying oven.

### 2.2. Preparation of Gelatin Solutions with Acid

Gelatin solutions with four concentrations (1.5, 2.0, 4.0 and 6.67%, *w*/*w*) were prepared. Firstly, accurately weighed gelatin was swelled in distilled water (1 h, ambient temperature), followed by being heated at 50 °C for 30 min with stirring (170 rpm). Next, analytical grade malic acid (MA), citric acid (CA), or lactic acid (LA) (0.5, 1.0 and 2.0%, *w*/*w*; all from Sigma-Aldrich) was added into a gelatin solution, and further heated at 50 °C for 30 min while stirring (170 rpm). The gelatin solutions were immediately used for the measurements of their pH values and viscoelastic properties. 

### 2.3. Measurement of pH 

The pH of gelatin solutions was measured following the protocol of Gelatin Manufacturers Institute of America [23] with a pH meter (PHS-3C, Shanghai Lei Ci Ltd., Shanghai, China). A two-point calibration was performed by using pH 4.00 and pH 6.68 buffers according to the pH-meter instructions. Determinations were carried out in triplicates for each sample, and the mean values were given.

### 2.4. Gel Strength

The strength of a gelatin gel (6.67%) with or without acid was measured based on the standard method of GMIA [23]. In short, gelatin (7.5 g) was added into a Bloom jar containing 105 mL of deionized water, then let stand at ambient temperature for 2 h with frequent stirring. Next, the sample mixture was heated in a water bath (65 °C) for 15 min with magnetic stirring, mixed with MA, CA, or LA so that the final gel contained 0.5–2.0% of acid, and cooled at ambient temperature for 15 min. Following this, the gelatin solution in the Bloom jar was kept in a refrigerator at 10 °C for 17 h, and used immediately for a gel strength test with a Texture Analyzer (TA-XT Plus; Stable Micro System, Surrey, UK) equipped with a 5 kN load cell and a standard plunger (diameter 12.70 mm). The gel was penetrated by the plunger at the speed of 0.5 mm/s for a distance of 4 mm, and the recorded force (g) corresponding to the 4 mm penetration was the gel strength (Bloom, g). Triplicate tests were performed for the gels obtained from independent preparations. 

### 2.5. Viscoelastic Properties

A Physica MCR 301 rotational rheometer (Anton Paar Physica, Graz, Austria) equipped with a cup-and-bob (rotor: diameter 26.7 mm; length 40 mm; cup diameter 28.9 mm) was applied in order to perform the dynamic studies. The controlled strain applied was 0.5% and the frequency used was 1 Hz so that the dynamic rheological tests were conducted within the linear viscoelastic region of the gelatin gels, based on some preliminary experiments. In order to perform a dynamic rheology test, a gelatin solution (2.2) was transferred into the sample cup, held at 40 °C for 10 min, cooled to 2 °C at a rate of 0.5 °C/min, held at 2 °C for 10 min, then heated back to 40 °C at the same rate, and held for 10 min. The changes of elastic (G′) and viscous (G″) moduli were collected during the temperature ramp test. The gelling temperature (T_G_) or melting temperature (T_M_) was defined as where G′ and G″ curves were crossed over for the first time during the cooling or heating processes [24]. All sample measurements were implemented in duplicate.

### 2.6. Statistical Analysis

Analysis of variance (ANOVA) was performed in order to evaluate whether the addition of LA, CA, or MA resulted in significant differences (*p* < 0.05) in the strength of gelatin gels. This was followed by a multiple pair comparison with the Tukey test if significant difference was found. The ANOVA and other data analysis (such as model fitting) presented in this study were conducted with SPSS 20.0 (Chicago, IL, USA).

## 3. Results and Discussion

### 3.1. Influences of LA, CA, and MA on the pH of Gelatin Gels

**Figure 1:**
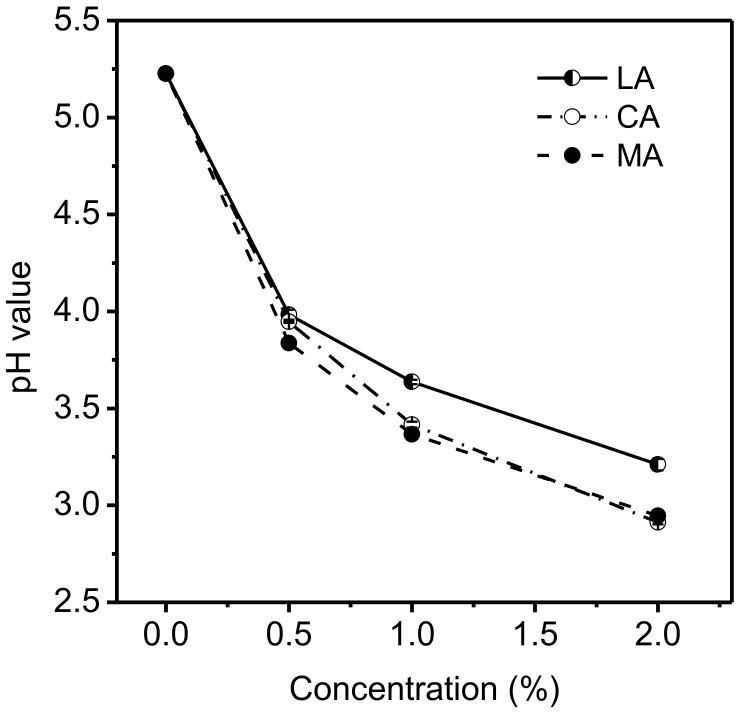
Effects of various concentrations of acids on the pH value of 6.67% gelatin solutions.

The changes of pH values after acidulants were added to the 6.67% gelatin solutions are shown in Figure 1. The addition of MA and CA at the same concentration in general resulted in no obvious difference in the pH of the gelatin solutions containing two different acids, but led to lower pH levels than those containing LA. Even with 0.5% of acid added, the pH of the gelatin solution decreased sharply, from 5.2 to 3.7 for MA or to 3.9 for CA and LA. When the acid concentration increased to 1.0%, the decrease in the pH value became slower, and the final pH for the gelatin solutions with acids were 3.3 (MA), 3.4 (CA), and 3.6 (LA), respectively. A further increase in the acid to 2% resulted in a small decrease in pH, to 3.2 for LA and to 2.9 for MA and CA. This behavior agreed well with the fact that the greatest buffering capacity of the system showed at pH values near its *pK_a_*, which was consistent with the related literature [25]. LA (*pK_a_* = 3.86), MA (*pK_a1_* = 3.46; *pK_a2_* = 5.05), and CA (*pK_a1_* = 3.13; *pK_a2_* = 4.76; *pK_a3_* = 6.40) are monobasic, binary, and triprotic acids, respectively (Table 1), and the dissociation of the second and third acid groups were considered to be negligible based on equilibrium constants.

### 3.2. Gel Strength

**Figure 2:**
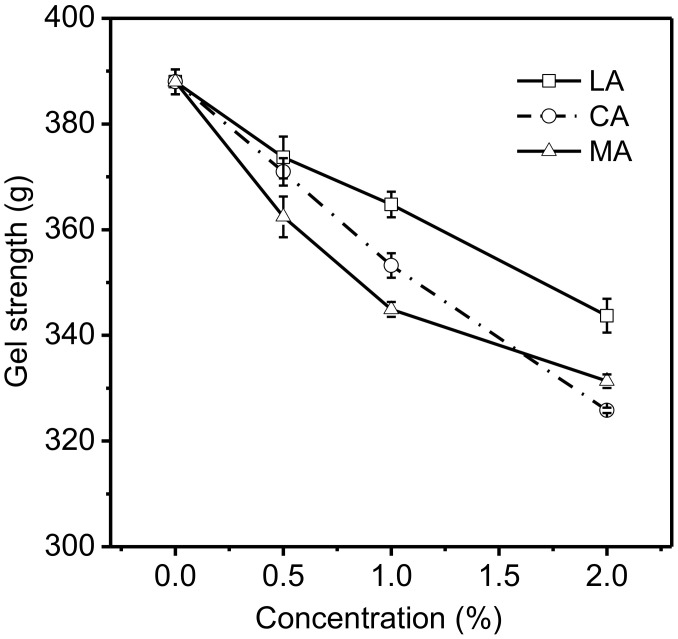
Effects of various concentrations of acids on the strength of 6.67% gelatin gels.

Gel strength is an important physical parameter which is used to evaluate the grade level and quality of gelatin [26]. The effects of various concentrations of acids on the strength of 6.67% gelatin gels are shown in Figure 2. The addition of LA, CA, or MA in gelatin gels had significant effects (*p* < 0.05) on their gel strengths. The gel strength of gelatin decreased when an increased level of LA, CA, or MA was added. Depending on the acid type, the introduction of 0.5% of acid led to a 14.3–25.5 decrease in the strength of gelatin gels, while the introduction of 1% and 2% of acid resulted in 23.2–43.1 and 44.3–62.2 decreases in the gel strength, respectively. The impact of the three acids on the strength of gelatin gels followed similar patterns to that on the pH of gelatin solutions. The gelatin gels with LA (1% or 2%) added had significantly (*p* < 0.05) higher gel strength compared to those with CA or MA at the same level, and the corresponding pH levels of gelatin solutions with LA were higher than those with CA or MA. The results were consistent with those reported by Choi [22], which indicated that the gel strength of porcine and fish gelatin decreased with the increase in acid added (pH = 2–4). Moreover, the study of Verheul [27] showed that the addition of acid could partially destruct the network of gelatin molecules, leading to the reduction in triple helical concentration. The addition of LA, CA, or MA into gelatin solutions resulted in the presence of more protons in the aqueous media, promoting the protonation of amino acid residues in gelatin. The protonation of amino acid residues in gelatin could negatively affect the formation of hydrogen bonds, which plays a critical role in forming the framework of gelatin gel [21,28]. Therefore, the strength of gelatin gels was generally weakened at a lower pH level.

### 3.3. Viscoelastic Properties

**Figure 3:**
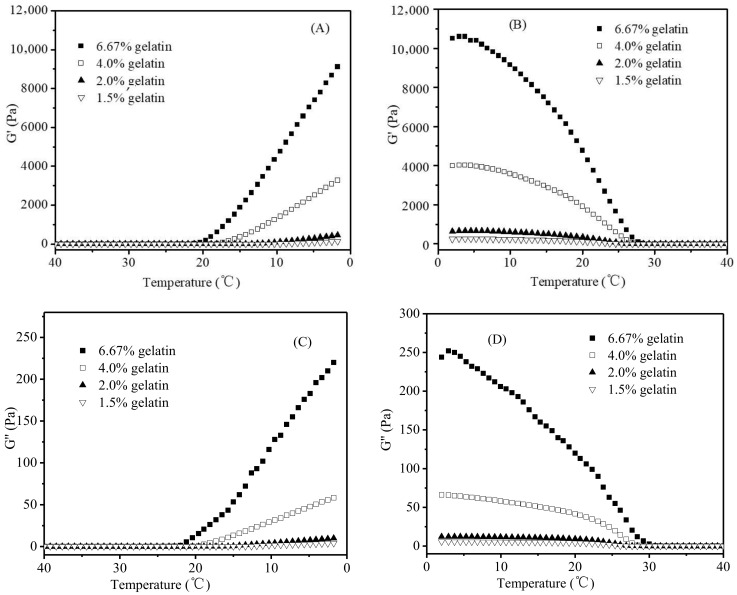
Changes of G′ and G″ of gelatin dispersions varying in the gelatin concentrations during the cooling stage (**A**/**C**), and heating stage (**B**/**D**).

Figure 3 shows the changes of G′ and G″ during the cooling (from 40 to 2 °C) and subsequent heating stages (from 2 to 40 °C) of gelatin dispersions varying in gelatin concentrations (1.5%, 2.0%, 4.0%, and 6.67%). Both G′ and G″ increased during the cooling stage (Figure 3A,C), and G′ increased more quickly, leading to a cross-over of G′ and G″. The cross-over could be explained as the formation of helices acting as cross-links to construct a three-dimensional branched network, leading to the sol-gel transition of a gelatin dispersion, which was consistent with the observations of other similar studies in the literature [20,29,30,31]. During the heating stage, G′ decreased more quickly than G″ with the increase in the temperature (Figure 3B,D), which was due to the melting of junction zones and the decrease in helical structures concentration [28], resulting in the gel-sol transition of gelatin dispersion. 

**Figure 4:**
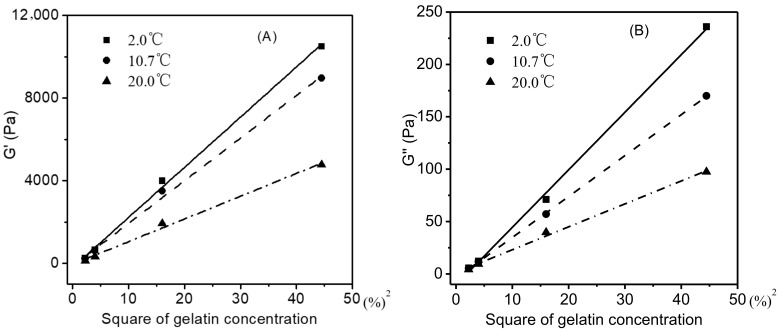
Square of gelatin concentration versus G′ of gelatin gels (2–20 °C) (**A**), G″(**B**).

A gelatin gel (≤20 °C) with a higher gelatin concentration corresponded to higher levels of G′ and G″ (Figure 3), which was attributed to its higher concentration of helices and junction zones of the gelatin network [32,33]. As shown in Figure 4A (only the data for the gelatin gels heated from 2 °C to the melting was shown here), when the G′ values of gelatin gels at each of the three representative temperatures (2.0, 10.7, and 20.0 °C) were plotted against the square of gelatin concentrations, a good linear relationship was observed (*R^2^* = 0.9981, 0.9971, and 0.9959, respectively). A similar relationship also existed between G′’ values and the square of gelatin concentrations (Figure 4B) (*R^2^* = 0.9983, 0.9999, and 0.9968, respectively). 

**Figure 5:**
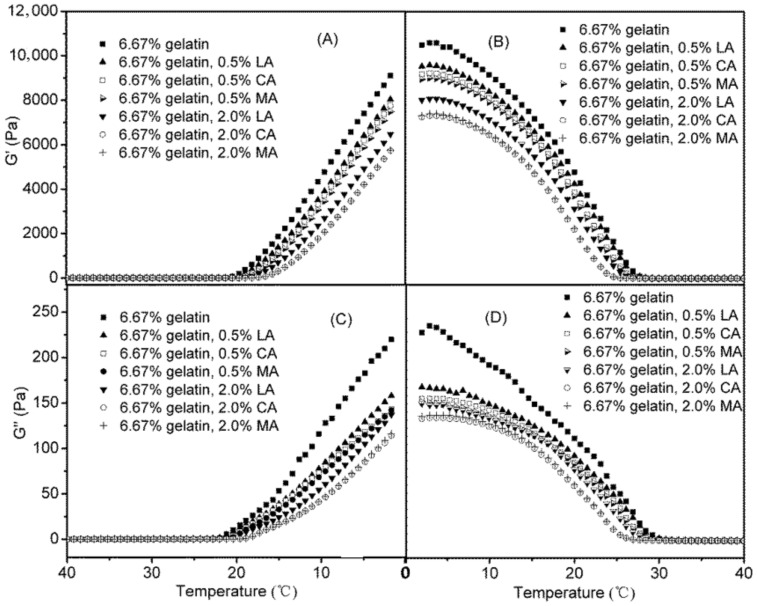
Changes of G′ and G″ of 6.67% gelatin dispersions, as affected by the addition of LA, CA and MA during the cooling (**A**/**C**) and heating stages(**B**/**D**).

Figure 5 shows the changes of G′ and G″ during the cooling and subsequent heating stages of representative gelatin dispersions (using 6.67% gelatin dispersions as examples) as affected by the addition of LA, CA, and MA (0.5% and 2.0%). The rheological properties of gelatin dispersions were greatly affected by the acid added, depending on the acid type and concentration. Both G′ and G″ of gelatin dispersions decreased in the presence of acids, and a higher acid concentration resulted in lower G′ and G″ values. The G′ and G″ values of gelatin dispersions were decreased to the furthest extent with the addition of 2.0% MA and CA, which were found to be almost identical. The addition of LA had less effect on both G′ and G″ values of gelatin dispersions as compared to that of MA and CA, which showed similar trends as the effects of acids on the pH of gelatin dispersions (Figure 1). MA and CA had a stronger ability to reduce pH at equal concentration compared with LA (Figure 1). Lowering the pH of a gelatin dispersion could promote the protonation of amino acid residues, which consequently interrupt the formation of hydrogen bonds and junction zones of the gelatin network, resulting in reduced values of G′, a measurement for elastically stored energy [21,27,28].

The rigidities of gels can be expressed with an empirical equation [34] as follows:

(1) $$\sqrt{G}=0.00484\left(\mathrm{Mw}-1.20\times 10^{10}e^{-\frac{7830}{\mathrm{RT}}}\right)$$

where Mw is the average molecular weight (g), T is absolute temperature(K), and G is the rigidity of a gel (N/m), which only slightly differs from G′ [35]. Based upon the equation, in a certain gelatin concentration (such as 6.67%), higher Mw leads to a higher G′ (or G″).

**Figure 6:**
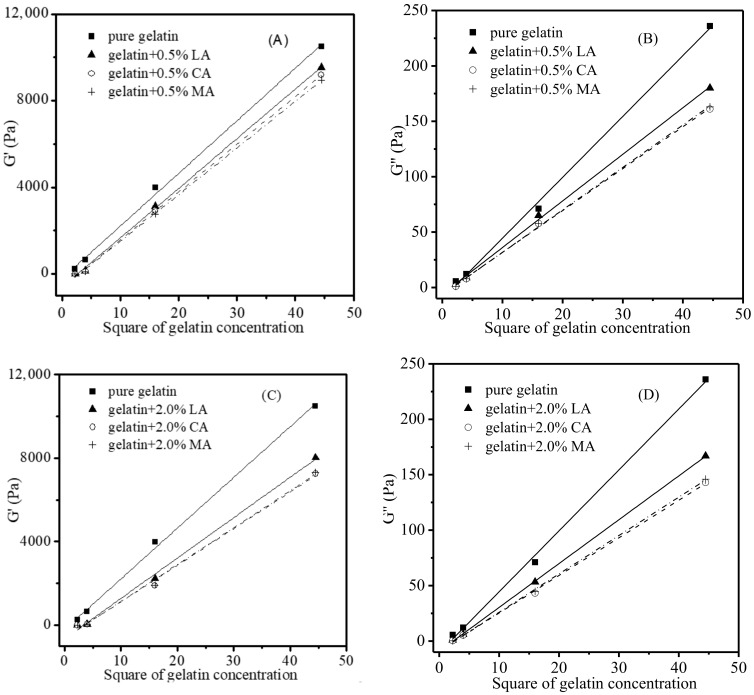
G′ and G″ of gelatin gels (2 °C) with different levels of LA, CA and MA versus square of gelatin concentrations. G′—0.5% (**A**), G′—2.0% (**B**), G″—0.5% (**C**), G″—2.0% (**D**).

Similarly, for the gelatin gels with no acid added (Figure 4), linear relationships were also discovered when the G′ and G″ of gelatin gels were plotted against the square of gelatin concentrations after the acids were introduced. As illustrated in Figure 6, the effects of acid concentration (0.5% and 2.0%) on the G′ and G″ of different gelatin concentrations (1.5, 2.0, 4.0, and 6.67%) at a specific temperature (2 °C) could be estimated via the aforementioned linear relationships. It is therefore possible to calculate the G′ and G″ of gelatin gels containing different amounts of gelatin (such as 3%) and that of gelatin gels added with other levels (such as 0.8%) of acid based on these relationships.

### 3.4. Gelling and Melting Temperatures

Table 2 shows the T_G_ and T_M_ of gelatin dispersions with different gelatin concentrations as affected by the addition of LA, CA, and MA. For the control without added acid, the formation of gelatin gels occurred at 21.8 °C (6.67% gelatin), 19.5 °C (4% gelatin), 14.8 °C (2% gelatin), and 11.8 °C (1.5% gelatin), while the corresponding melting temperatures were 29.3 °C, 28.2 °C, 26.9 °C, and 26.0 °C, respectively. This clearly showed that the higher the gelatin concentration was, the higher the T_G_ and T_M_ of the gelatin dispersion were, which was consistent with the results reported by Joly-Duhamel [33]. A higher concentration of gelatin resulted in the formation of higher levels of helices and junction zones in the aqueous media, as well as shorter distances between junction zones, leading to the formation of a stronger network within the gelatin dispersion [36,37]. 

**Figure 7:**
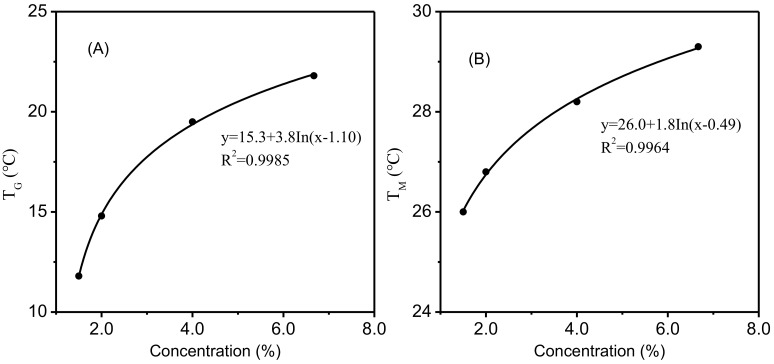
The relationship between gelatin concentration and T_G_ (**A**), T_M_ (**B**).

**Figure 8:**
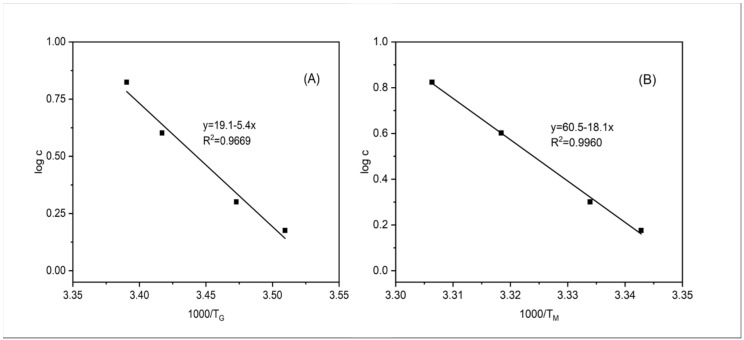
The relationship between the logarithm of gelatin concentration and T_G_ (**A**), T_M_ (**B**).

The relationship between gelatin concentration and gelling temperature or melting temperature agreed fairly well in a logarithmic model (Figure 7) (*R^2^* = 0.9985 or 0.9964). Eldridge and Ferry [38] developed a mathematical model to relate the melting temperature of gelatin gel to its gelatin concentration as follows:

(2) $${\mathrm{Log}}_{10}c=\frac{\Delta H^{0}}{2.303\mathrm{RT}}+\mathrm{constant}$$

where c is the gelatin concentration (g/L), T is absolute temperature of melting (K), R is the gas constant (8.314 J/mol.K), and ΔH^0^ is the melting enthalpy (J/mol). A plot of the logarithm of gelatin concentrations against the reciprocal of the melting temperatures (K) based upon the equation (2) showed a good linear relationship (*R^2^* = 0.9669, Figure 8A), indicating that the empirical model worked quite well for the fish gelatin gels in this study. This model, developed by Eldridge and Ferry [38], has been widely applied in many other polymer gels such as gellan gels [39], agarose gels [40], and k-carrageenan gels [41], as well as gelatin gels with polyols and sugars added [42]. Moreover, a good linear relationship was also found between the reciprocal of gelling temperature (K) and the logarithm of gelatin concentrations (*R^2^* = 0.9960, Figure 8B). These models could be used to calculate the gelling and melting temperatures of gelatin dispersions with gelatin concentrations not included in this study (such as those containing 0.8% gelatin).

In the presence of 0.5–2.0% of LA, CA, or MA, both T_G_ and T_M_ of gelatin dispersions (2.0–6.67% gelatin) decreased (Table 2). For the 6.67% gelatin dispersions, the addition of LA, CA, or MA resulted in the decrease in T_G_ from 21.8 °C to 20.2–21.4 °C (LA), 18.7–20.4 °C (CA), or 18.7–20.3 °C (MA), depending on the concentration of the acid added. For the 4.0% gelatin dispersions, the corresponding T_G_ decreased from 19.5 °C to 15.4–17.4 °C (LA), 13.3–16.4 °C (CA), or 13.3–16.1 °C (MA). For the 2.0% gelatin dispersions, the corresponding T_G_ decreased from 14.8 °C to 3.5–7.7 °C (LA), 3.1–6.5 °C (CA), or 2.8–6.1 °C (MA). Noticeably, a higher level of acid added resulted in a lower T_G_ of the gelatin dispersion, while the effects of CA and MA were more obvious than those of LA at the same level, which was tied to the pH of the final gelatin dispersions (Figure 1). Moreover, the reduction in T_G_ for gelatin dispersions with a low gelatin concentration (such as 2.0%) due to the addition of an acid was greater than that with a high gelatin concentration (such as 6.67%). For example, the T_G_ dropped only 0.4 °C (from 21.8 to 21.4 °C) for the 6.67% gelatin dispersion due to the addition of 0.5% LA, but dropped 2.1 °C (from 19.5 to 17.4 °C) for the 4.0% gelatin dispersion and 7.1 °C (from 14.8 to 7.7 °C) for the 2.0% gelatin dispersion. The effects of LA, CA, and MA on the T_M_ of gelatin dispersions (2.0–6.67%) follow similar patterns as that on T_G_ (Table 2). The T_M_ decreased from 29.3 °C to 28.8–29.1°C (LA), 26.8–28.4 °C (CA), or 26.8–28.2 °C (MA) for the 6.77% gelatin dispersions with the addition of an acid, while it decreased from 28.2 °C to 26.1–27.6 °C (LA), 24.3–26.7 °C (CA), or 24.5–26.6 °C (MA) for the 4.0% gelatin dispersions, and from 26.8 °C to 22.7–25.2 °C (LA), 21.9–24.4 °C (CA), or 22.0–24.4 °C (MA) for the 2.0% gelatin dispersions. For the dispersions containing 1.5% gelatin and acid, there was generally no G′/G″ cross-over observed, except for that with 0.5% or 1.0% LA. Therefore, no T_G_ and T_M_ were reported. 

The gelling and melting temperature of a gelatin dispersion reflected the transition temperatures of the coil–helix of the dispersion system [43]. The decreases in T_G_ and T_M_ of gelatin dispersions due to the addition of LA, CA, or MA were attributed to the effects of the acids on interrupting the formation of hydrogen bonds and weakening the junction zones of the gelatin molecules, which were similar to the effects of acids on the strength of gelatin gels and the G′ and G″ of gelatin dispersions [21,27,28]. Regarding the less pronounced effects of LA, CA, and MA on the T_G_ and T_M_ of a dispersion with higher gelatin content, this could probably be attributed to the stronger buffering effect of the dispersion. In addition, since the number of junction zones within the network of a dispersion containing higher gelatin content was much higher than in that with low gelatin content, the percentage reduction in the junction zones in the high gelatin dispersion due to the addition of an acid would be relatively less.

## 4. Conclusions

The addition of LA, CA, or MA in gelatin dispersions greatly affected the gel strength, G′, G″, and phase transition temperatures, including T_G_ and T_M_. The gel strength, G′, G″, T_G_, and T_M_ of gelatin dispersions decreased with an increased level of LA, CA, or MA added, indicating the weakening effects of these acids on junction zones of the gelatin network in aqueous media. In addition, these rheological properties of gelatin dispersions were less affected by LA as compared to MA and CA, which were consistent with their effects on the pH of gelatin dispersions. Furthermore, the influences of an acid on the T_G_ and T_M_ of a dispersion with higher gelatin concentration were less pronounced compared to on a dispersion with lower gelatin content, which could be attributed to the stronger buffering effect of the high gelatin dispersion and less percentage reduction in the junction zones in the dispersion due to the addition of an acid. There were linear relationships between the square of gelatin concentrations and G′ (*R^2^* = 0.9959–0.9981) or G″ (*R^2^* = 0.9968–0.9999) at a specific temperature, and similar relationships also existed for the gelatin dispersion with an acid. Therefore, it is possible to develop a model to calculate the G′ and G″ of gelatin dispersions containing different amounts of gelatin and acid based on these relationships, and to predict their phase transition temperatures, in the near future.

## Figures and Tables

**Table 1:** Chemical properties of lactic acid, malic acid, and citric acid.

Acidulant	Formula	Molecular wt (g/mol)	*pK_a1_*	*pK_a2_*	*pK_a3_*
Lactic acid	C_3_H_6_O_3_	90.08	3.86	—	—
Malic acid	C_4_H_6_O_5_	134.09	3.46	5.05	—
Citric acid	C_6_H_8_O_7_	192.12	3.13	4.76	6.40

Data acquired from the website: http://pubchem.ncbi.nlm.nih.gov (accessed on 20 January 2022) (NIH, 2022).

**Table 2:** The gelling (T_G_) and melting (T_M_) temperatures of tilapia skin gelatin dispersions (1.5–6.67% gelatin) as affected by the addition of lactic acid (LA), citric acid (CA), and malic acid (MA).

Acids		Control	LA			CA			MA		
Concentration			0.5%	1.0%	2.0%	0.5%	1.0%	2.0%	0.5%	1.0%	2.0%
6.67% gelatin	T_G_	21.8	21.4	21.0	20.2	20.4	19.5	18.7	20.3	19.4	18.7
	T_M_	29.3	29.1	29.0	28.8	28.4	27.7	26.8	28.2	27.5	26.8
4.0% gelatin	T_G_	19.5	17.4	16.7	15.4	16.4	14.8	13.3	16.1	14.8	13.3
	T_M_	28.2	27.6	26.9	26.1	26.7	25.3	24.3	26.6	25.3	24.5
2.0% gelatin	T_G_	14.8	7.7	5.5	3.5	6.5	4.6	3.1	6.1	4.4	2.8
	T_M_	26.8	25.2	24.1	22.7	24.4	23.0	21.9	24.4	23.1	22.0
1.5% gelatin	T_G_	11.8	—	—	—	—	—	—	—	—	—
	T_M_	26.0	22.0	19.4	—	—	—	—	—	—	—

## Data Availability

Data is contained within the article.
